# Occurrence and management of premature clinical trial termination: a survey of UK healthcare professionals

**DOI:** 10.1186/s13063-026-09441-9

**Published:** 2026-01-22

**Authors:** Helen Pluess-Hall, Julie Menzies, Paula Smith

**Affiliations:** 1 Department for Health, University of Bath, Bath, BA2 7AY UK; 2 University Hospitals of Bristol and Weston NHS Foundation Trust, Bristol, UK; 3 University of Bath, Bath, UK

**Keywords:** Clinical research, Trial termination, Healthcare professionals, Premature termination, Research delivery

## Abstract

**Background:**

A proportion of clinical trials terminate prematurely, due to logistical or conduct issues and emerging scientific data. Due to a paucity of literature and non-standardised reporting, the rate for all trials is unknown, and little is known about the number and experience of healthcare professionals managing this situation. This study aimed to identify how many UK healthcare professionals delivering clinical research have experienced premature trial termination**,** the challenges experienced and resources available for managing this situation.

**Methods:**

Following ethics approval, a national e-survey of self-identifying healthcare professionals delivering clinical research was conducted (April–September 2022). Analysis included descriptive statistics and content analysis for categorization of challenges.

**Results:**

A total of 65% (*n* = 89) of healthcare professionals had experienced ≥ 1 premature trial termination. Challenges included communicating with research participants and/or families (*n* = 20) and emotional distress for participants and/or families (*n* = 21). Forty-eight healthcare professionals identified resources; of those available for review, one resource provided guidance relating to research participants.

**Conclusion:**

Premature clinical trial termination creates challenges for care delivery and impacts on participants and/or families. Healthcare professionals need preparation and training to ensure participants are appropriately supported if their trial prematurely terminates.

**Supplementary Information:**

The online version contains supplementary material available at 10.1186/s13063-026-09441-9.

## Key points

- The majority of UK healthcare professionals have experienced clinical trials prematurely terminating.
- Healthcare professionals reported experiencing challenges and perceived that research participants and/or families also found the situation to be challenging. Challenges were both emotional and practical in nature.
- Healthcare professionals have a lack of formal resources to help them manage participants in the event of a clinical trial prematurely ending.
- Healthcare professionals, research participants and families need to be aware of this potential situation.
- Future research is needed to explore the experience and what resources healthcare professionals find helpful in supporting this situation.

**Supplementary Information:**

The online version contains supplementary material available at 10.1186/s13063-026-09441-9.

## Background

Clinical trials evaluate the effect interventions have on human health outcomes [1]. Individuals participating in clinical research may derive a direct clinical benefit and the opportunity to access treatments otherwise unavailable. The National Health Service in the UK recognises the benefits research brings for participants and patients within research-active hospitals, most notably lower mortality rates [2]. The hope is that research will identify new treatments or methods that improve outcomes, but it is also valuable to demonstrate ineffectiveness and discontinue those treatments. A proportion of trials terminate prematurely before they have enrolled and treated the anticipated number of participants. The overall premature termination rate is unknown due to a paucity of literature and non-standardised reporting. In oncology trials, there is a reported rate of 23% for adults [3] and an estimated 10% for paediatric trials across all specialities [4]. In addition to non-standardised reporting when trials end, there is also variation in the terminology used to describe trials that stop earlier than originally planned. The terms premature termination, early termination, early end and termination can and are used interchangeably in clinical practice and the literature. Premature termination is the preferred term for this paper as it is in line with national guidance [5] and the term used throughout the project.

Trials can be terminated for many reasons, including logistical or conduct issues and due to scientific data generated by the study [6]. Patient safety is a key reason for premature termination; however, other reasons include the most common reason of insufficient recruitment of participants and unspecified business decisions, but not all trials record a reason [6–8]. Trial data is continuously reviewed, and if the intervention is showing a clear benefit, no effect or unacceptable adverse events, the trial will be terminated. In trials showing clear benefit, research participants may continue to receive the intervention after the trial is stopped; however, participants within trials prematurely terminated for ineffectiveness or safety issues may have their trial treatment or medication abruptly stopped. Healthcare professionals delivering the research are then required to manage the emotional and practical consequences for the participant and their family. In these cases, premature trial termination has been shown to be negatively experienced, resulting in parents who felt distressed, unprepared and with a loss of engagement [9]. Similarly, adult participants of one trial felt frustrated and fearful with the communication they received about the trial termination whilst waiting to be unblinded to their treatment allocation [10].

A review of paediatric clinical trial protocols found that whilst the majority of protocols acknowledged the potential for premature termination, 58.3% did not include detail on providing participant management or care in this situation [11]. It is not known what other resources may be available to support the management of this situation.

This study aimed as follows:

1. Identify how many healthcare professionals involved in clinical research delivery have experienced the premature termination of a clinical trial.
2. Identify challenges healthcare professionals experienced when trials prematurely terminated.
3. Identify healthcare professionals’ views on the challenges participants and families experience when trials are prematurely terminated.
4. Identify and review the resources healthcare professionals use to manage the premature termination of clinical trials.

## Methods

### Study design

A national cross-sectional survey of UK healthcare professionals delivering clinical research as part of their role was conducted (April–September 2022) to scope the proportion who had experienced the premature termination of a clinical trial and identify challenges and resources available to manage the situation.

A Consensus-Based Checklist for Reporting of Survey Studies (CROSS) [12] was used to guide reporting.

### Participants

This study included UK healthcare professionals delivering clinical research within their role who consented to take part. Participants self-determined if they met the eligibility criteria.

Participants were recruited using purposive and snowball sampling [13]. The study advert, including the link to the online questionnaire, was advertised using social media and promoted within the researchers’ existing professional networks of healthcare professionals supporting and delivering research, including at the Royal College of Nursing International Nursing Research Conference 2022. To ensure as wide a participation as possible, access to the survey was open, meaning it could be completed without receiving a specific invitation link or using a designated email address.

Calculating a denominator was challenging as this was a scoping survey of an undefined population. The number of UK clinical research nurses and midwives was known (*n*= 7469) from a self-reporting survey supported by the National Institute for Health and Care Research (NIHR), which also acknowledged the challenge when scoping a workforce of undefined size [14]. Therefore, whilst the actual denominator cannot be reported, it was at a minimum 7469.

### Data collection

An electronic questionnaire was chosen as an appropriate data collection tool as it enabled standardised questions to be asked to all participants, facilitated participation from healthcare professionals across the UK and minimised the research burden for participants by allowing data to be provided quickly at a time suitable for them [15]. As healthcare professionals are required to have technology literacy in order to fulfil their clinical roles [16], they would be able to successfully engage with the format.

No validated tool existed; therefore, a questionnaire was designed and created for this study in Jisc (supplemental material). The questionnaire utilised a branching structure with the maximum number of questions that could be answered being 15. Questions were related to identifying the trial characteristics, challenges experienced and available resources. It was piloted with a group of 17 research nurses of varying clinical backgrounds, seniority and experience. No issues were identified with the functionality of the electronic questionnaire; it was established that the questions were not being misinterpreted and therefore eliciting the intended data, but minor formatting and spelling corrections were made. As the pilot successfully demonstrated, the questionnaire was functional; there was no other pilot after the minor changes were made.

The questionnaire collected anonymous responses, with an optional unlinked questionnaire on completion capturing identifiable details for the purpose of a prize draw (1 × £50 shopping voucher) and future research-related contact. Collecting anonymous data allowed participants to describe challenges without concern for breaking confidentiality and granted the freedom to discuss services without reputational concern. The identifiable information was kept confidentially, stored securely on the university server and deleted once used for the purpose(s) for which consent had been provided.

### Data analysis

Data from those who did not meet the inclusion criteria were excluded from the analysis.

Data was exported from Jisc into Microsoft Excel. As a short anonymous survey of both open and closed questions, data cleaning consisted of reviewing the free-text answers to the open-ended questions to check for duplicates, ensure they could be understood, and that the answers were relevant to the question asked. Eight answers lacked enough information to understand the meaning. Sixteen answers were irrelevant to the question being asked, where the answer was relevant to another question that data was transferred to be included in the analysis of that question. Eight answers described challenges that led to the trial prematurely terminating, rather than challenges resulting from the premature termination. No identical answers were present, and where participants chose to provide personal details, there were no duplicates. Jisc does not capture the data provided in any partial, incomplete or unfinished questionnaire responses; therefore, there were no missing data points.

Descriptive statistics were used with the quantitative data to quantify the proportion of participants who had experienced this phenomenon and how many had guidance in place.

The qualitative data related to challenges experienced were analysed using qualitative and quantitative content analysis [17, 18] to produce categories and frequency data. An inductive approach was taken due to little existing literature on this topic; the coding framework evolved during the analysis. Coding was predominately manifest, due to the nature of the data being short specific answers to the survey questions.

Resources were reviewed to identify information relating to premature termination. This was then compared to the identified issues resulting from the qualitative survey data to determine if the guidance was relevant to the healthcare professionals’ needs.

Data cleaning and analysis were undertaken by HPH. Research supervisors J. M. and P. S. were co-coders [19], and discussion within the team was added to the interpretation of the data.

## Results

**Fig. 1:**
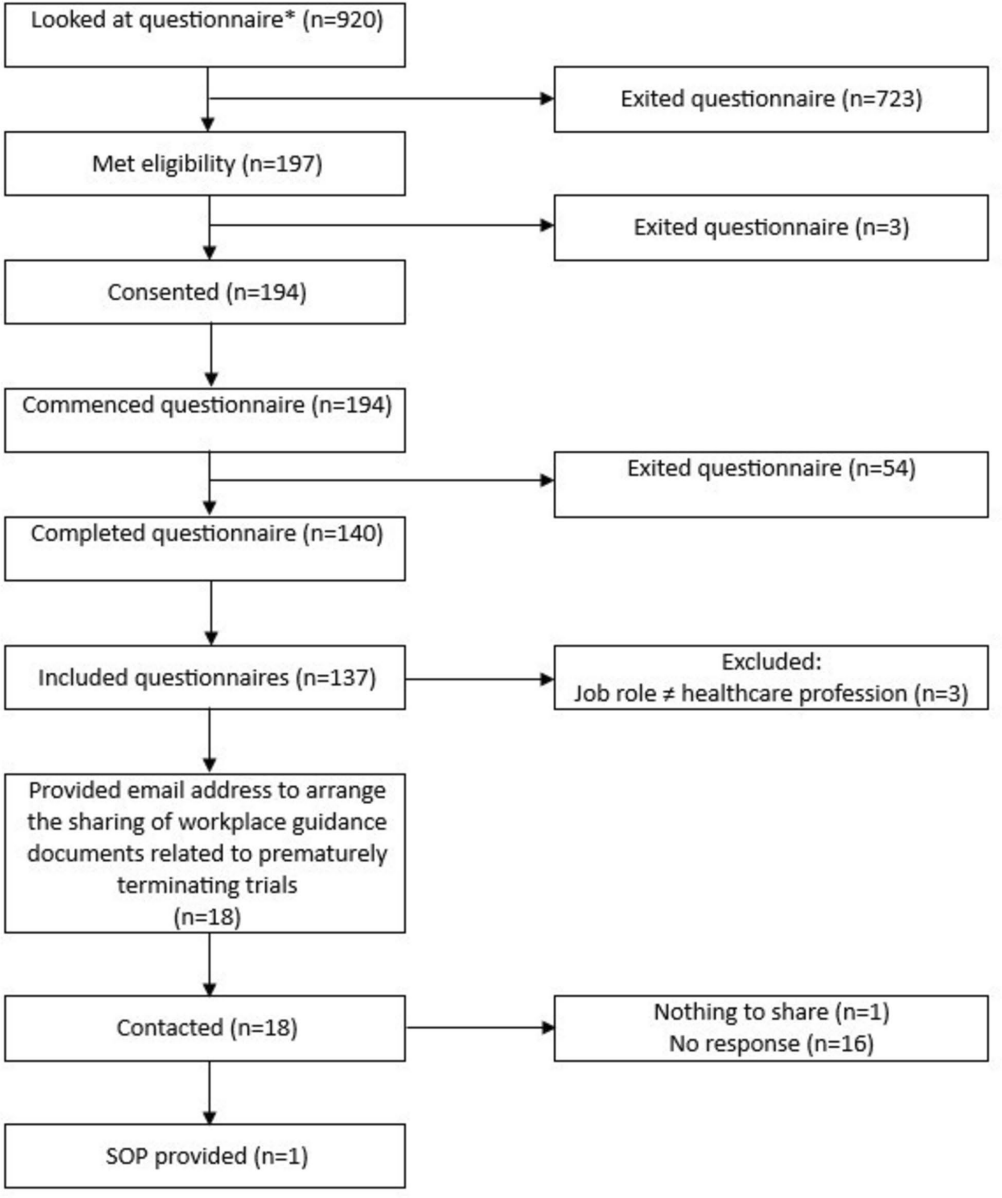
Prisma diagram. *Minimum denominator = 7469

The questionnaire was opened by 920 people, with 709 opening the first page and not continuing further and 194 consenting to participate. One hundred forty complete responses were received; therefore, the study completion rate was 72.2%. We are unable to report a response rate, as both the target population size and the number of potential participants aware of the study are unknown. Three complete questionnaires were excluded, as they reported their profession as ‘other’ in reference to the list of healthcare professionals offered and reported a job role that did not meet the inclusion criteria. A total of 137 complete questionnaires remained and were included in the analysis (Fig. 1).

For clarity, the participants of this study will be referred to as ‘respondents’ or ‘healthcare professionals’, and the term ‘participants’ will be used for those who took part in the clinical trials that prematurely terminated.

The respondents reflected a variety of healthcare professions, with the majority being from nursing (Table 1).

**Table 1:** Questionnaire respondents’ profession

Profession	Proportion of respondents % (*n* =)
Nurse	66.4% (*n* = 91)
Doctor	14.6% (*n* = 20)
Midwife	6% (*n* = 8)
Clinical research practitioner	2% (*n* = 3)
Radiographer	2% (*n* = 3)
Physiotherapist	1% (*n* = 2)
Pharmacist	1% (*n* = 2)
Occupational therapist	1% (*n* = 2)
Pharmacy technician	1% (*n* = 2)
Dietician	1% (*n* = 1)
Psychologist	1% (*n* = 1)
Assistant research practitioner	1% (*n* = 1)
Clinical trials assistant	1% (*n* = 1)

Nearly all respondents (99%, *n* = 135) worked within the NHS, and 65% (*n* = 89) had experience of working on at least one trial that had prematurely terminated. Healthcare professionals had experienced the premature termination of trials with adult and paediatric participants (Table 2), and 71.9% (*n* = 64) experienced premature termination resulting in a medication, therapy, device usage or treatment being stopped.

**Table 2:** Population of the prematurely terminated clinical trials

Trial participants	Proportion of UK healthcare professionals who had experienced prematurely terminated trials by participant population % (*n* =)
Adult	60.7% (*n* = 54)
Paediatric	34.8% (*n* = 31)
Both adult and paediatrics	4.5% (*n* = 4)

A total of 70% (*n* = 62) of healthcare professionals experienced challenges personally or for the service, and 60.7% (*n* = 54) reported challenges for participants and/or families. Thirty-two issues were reported for healthcare professionals or the service, and thirty-five issues were reported for participants and/or families. Of those who reported no issues for participants and/or families (20.4%, *n* = 28), 21.4% (*n* = 6) qualified this as due to not having current participants either at all or in the treatment phase when the study prematurely terminated. One could not comment on the participants’/families’ issues posttrial termination as they were followed up by another team.

### Overview of challenges

There was commonality of challenges identified for both healthcare professionals and the service and participants and/or families (Table 3).

**Table 3:** Overview of challenges identified by UK healthcare professionals

**Categories**	**Subcategories**	**Healthcare professionals or the service** (*n* = no. of healthcare professionals reporting challenge)	**Participants and/or families** (*n* = no. of healthcare professionals reporting challenge)
Communication		25	0
Emotional distress		10	21
Support	Supporting participants and/or families	7	0
	Lack of support	0	2
	Losing research team support	0	5
Practical issues	Transitioning participants to standard care	6	4
	Staff time	11	0
	Wasted resources	10	0
Information	Lack of information	11	4
	Information sharing method	2	0
Research value		3	0
Personal health	Perceived benefit of investigational medicine	0	4
	Concern for health	0	12

### Challenges

We report results of the qualitative content analysis in the continuous text style, organised by category and include direct quotes illustratively within the category descriptions [20]. We have included the number of healthcare professionals reporting the issue and what percentage that is of the total number of healthcare professionals who had experienced premature trial termination (*n* = 89).

#### Communication

Communicating with participants and/or families was the most reported challenge for healthcare professionals (*n* = 20, 22.5%). It encompassed a range of specific situations that healthcare professionals found to be challenging: explaining why the trial was terminating prematurely and that the study treatment was no longer available and answering questions and scenarios where the healthcare professional felt they were not able to be entirely truthful. One example of this was parents not wanting the reason for the premature termination to be known by their child (the trial participant), which added a layer of complexity to the final research study visit.

This was very difficult news for the family, the trial was stopped early due to no positive results from an interim data lock - the drug was not seen to work, this was for a [rare disease] and may have been felt like a last chance to save their child, recruitment to the study was very competitive and this family were very grateful to get into the trial, it was emotional for parents and research nurses at the final study visit doing safety bloods etc, to say goodbye and we made sure it felt safe and comfortable for the child and parents hid the* real reasons for the trial ending from their child. (*Respondent 54)

An additional five healthcare professionals (5.6%) identified communication as an issue without detailing what the issue related to or with whom.

#### Emotional distress

Premature trial termination caused 10 healthcare professionals’ (11.2%) emotional distress, with 2 (2.2%) reporting multiple emotional responses including anxiety, frustration and concern.

Emotional distress was the most reported issue that healthcare professionals perceived participants and/or families to have experienced (*n* = 21, 23.6%). This encompassed loss of hope, anxiety, stress, disappointment and difficulty in accepting the situation.

Realisation that there wasn’t another treatment option and disappointment that treatment hadn’t worked. (Respondent 34)

#### Support

Seven (7.9%) reported that supporting participants and/or families through a challenging situation and managing their expectations were challenging for healthcare professionals.

It was difficult to manage as the patient believed to have good response to the treatment and it was only treatment so far targeted to delay progression of the disease. (Respondent 67)

Healthcare professionals identified that participants and/or families felt a lack of support (*n* = 2, 2.2%) and found losing the support of the research team challenging (*n* = 5, 5.6%).

#### Practical issues

Healthcare professionals (*n* = 13, 14.6%) reported 15 issues with the practicalities of prematurely terminating the trial (nonparticipant related). Short deadlines and the logistics of dealing with no longer required equipment and medication highlight the impact termination had on workload. This was reflected by 11 (12.4%) healthcare professionals raising the need for additional staff time as an issue.

Time and effort from all teams involved including governance and pharmacy. (Respondent 15)

Ten (11.2%) were challenged by the waste of resources which encompassed the waste perceived in setting up and opening the trial, approaching potential participants about the study and screening participants.

Practical issues related to participants included assisting with the transition of participants into standard of care (*n* = 6, 6.7%), with 3 (3.4%) of healthcare professionals highlighting that the treatment being trialled was the only treatment targeting that condition or the progression of a disease. Four (4.5%) reported that transitioning to care outside of the clinical trial was also challenging for participants and/or families.

Eleven healthcare professionals (12.4%) reported that participants and/or families experienced issues with the practicalities required when the trial prematurely terminated.

Attendance every week day for 8 weeks was a challenge (Respondent 20)

One healthcare professional identified complying with the rules and regulations governing clinical trial conduct was problematic for participants. An essential task is informing the research participants, and one healthcare professional highlighted that the participants being contacted urgently caused them to feel alarm.

It can be tricky getting hold of families urgently without panicking them if a message had to be left. (Respondent 59)

#### Information

Eleven healthcare professionals (12.4%) experienced a lack of information related to the termination rationale and practical concerns such as the next steps for participant follow-up and medication.

Two (2.2%) raised the way in which the information about the trial prematurely terminating was shared as an issue found to be incredibly challenging. The news was released into the public domain before being shared with healthcare professionals and participants. One healthcare professional reported families finding out before the research team could tell them, and one reported trying to rapidly contact families to inform them before they found out from another source.

…The drug company released information publicly that the trial was being stopped early, our team were lucky to have a colleague from the study who phoned us a fast as possible, we then tried to call the family before they heard it from anyone else (Respondent 54)

Four healthcare professionals (4.5%) reported that participants’ and/or families wanting more information as an issue.

#### Research value

Healthcare professionals (*n* = 4, 4.5%) were concerned that the premature termination of the trial devalued research in the eyes of colleagues and participants and worried they would be reluctant to participate in future research.

…The trial involved [procedure with critically ill patients] and so it had been challenging to engage staff. When the trial was stopped early, this was due to there being no patient benefit. The value and view of research on the unit by the clinical teams definitely took a knock… (Respondent 53)

#### Personal health

Four healthcare professionals (4.5%) reported that participants and/or families found the trial termination difficult as they believed that the medication or treatment they were receiving as part of the trial was effective, and that the loss of the trial would therefore directly impact their health.

…Loss of drug they thought was helpful. (Respondent 30)

Healthcare professionals (*n* = 7, 7.9%) identified the loss of a treatment option as a challenge experienced by participants and/or families, and 5 (5.6%) reported that participants and/or families were concerned about health: their health and overall survival now that the trial treatment was no longer an effective option, concerned that they may have been taking something within the trial that was detrimental to their health and concerned that the quality of care they were receiving would reduce now that they were outside of the trial.

### Managing premature trial termination

Thirty-five percent (*n* = 48) of all respondents reported having tools/strategies/standard operating procedures (SOPs) for managing premature trial termination (Table 4).

**Table 4:** Resources identified for managing premature trial termination

Description of resource	Number of healthcare professionals identifying the resource
Standard operating procedure (SOP)	15
Trial protocol	3
Process map	2
Research and Development Department	2
Intensive care	2
Clinical trial management System	2
X-ray	2
Risk assessment	1
Governance board	1
Research team	1
Midwives and nurses	1
Counselling	1
Study Clinical Research Associate (CRA)	1
Research or clinical staff that have previous experience	1
Study amendment documents	1

Of the identified resources, 15 SOPs, 2 process maps, and 1 risk assessment were considered to have the potential to guide healthcare professionals in managing the situation and suitable for content review. Thirteen percent (*n* = 18) of respondents were happy to be contacted to share resources and provided their email addresses for this purpose. All were emailed by the study team: 1 replied to say they had nothing to share, 1 supplied an NHS Trust SOP, and 16 did not respond. A maximum of three emails were sent to request resources. The details for one SOP were provided within a questionnaire response and were able to be located online. Upon review, the two SOPs clearly documented the regulatory requirements and procedures relating to the compilation and submission of study documentation and reports required at the end of the study. Premature termination was included, highlighting the shortened timeline for review boards to be informed and the responsibility of the chief investigator (CI) for submitting any required amendments to notify sites and to manage communication with participants. One SOP stated that participants must be fully informed about the implications of early termination and their choices in terms of post-research care. It describes how an unambiguous message should be prepared, approved and disseminated from CI to sites and then to participants. In relation to the challenges previously identified, the SOPs did not provide relevant guidance for healthcare participants managing the situation, particularly given the reality of the disorganised notification process regarding premature trial termination. Communicating information to participants was the one item that was both included in an SOP and identified as a challenge by healthcare professionals; however, the direction provided in respect to this in the SOP could not be followed without supplementary information from the trial sponsor.

## Discussion

This study investigated the experience of prematurely terminated clinical trials from the perspective of UK healthcare professionals. While other studies have reported premature termination rates for trials, the proportion of healthcare professionals who had experienced this was unknown. Very few studies have looked at the experience of a prematurely terminating trial, and this study adds novel insight into the challenges experienced by those delivering research. This study expands on previous work reviewing clinical trial protocols to include other guidance documents available to support healthcare professionals in managing the situation.

The overall premature termination rate is difficult to quantify, but it has been estimated that premature termination occurs in approximately 10% of paediatric trials [4] and is more common in trials with adult participants [3]. This study supports that this is a situation widely encountered by UK healthcare professionals delivering clinical research as part of their role, with 65% reporting experience of ≥ 1 clinical trial prematurely terminating. However, it is not a topic that features in the training available from NHS England [21] or the National Institute of Health and Care Research (NIHR) [22]. Most notably, it is not discussed within Good Clinical Practice (GCP) [23], the required training for researchers delivering clinical trials of investigational medicinal products (CTIMPs). Healthcare professionals will be required to manage the situation if the trials they are delivering prematurely terminate. Preparation and training for this eventuality should therefore feature in staff training.

To date, there has been little literature identifying the impact of premature trial termination for participants, families and healthcare professionals. New research published in the time since this study closed has continued to focus on the reasons for trial termination and quantifying premature termination rates rather than the experience [3, 24, 25]. This study has provided novel evidence, highlighting the personal or service challenges healthcare professionals experience and the perceived challenges for participants and families associated with premature trial termination. That this is a challenging experience is consistent with the two previous studies exploring premature termination [9, 10]. The most reported consequence of premature trial discontinuation was emotional distress of healthcare professionals, participants and/or families. This encompassed loss of hope, disappointment, frustration, anxiety, stress and difficulty in accepting the situation. These findings are consistent with adult cancer patients and carers contemplating clinical trials [26] and hope being a common reason for trial participation [27]. Ensuring healthcare professionals have the required skills to manage their own emotional wellbeing and support patients is important for all working in healthcare. Communicating the concept and potential of a trial prematurely terminating to participants and/or families throughout their research journey may lessen some of the associated emotions by reducing the element of surprise. Currently, the Health Research Authority Guidance [28] on developing patient information sheets and consent forms does not include premature termination. Participating in clinical research is not without burden [29], and this study shows that terminating participation does not immediately remove this; it is important to ensure that support for participants does not end abruptly if their clinical trial does.

One of the striking results of the study was the lack of tools, strategies or resources available to healthcare professionals for managing premature termination. Only 15 respondents cited the availability of a related SOP, suggesting a lack of formalised planning for managing participants in this situation. What is not known is if healthcare professionals have used the resources they identified and, if so, whether they found them to be helpful. Of the resources available for review (*n* = 2), only one addressed the challenge of information provision to participants’ and families. This suggests that the healthcare professionals lacked the information to be able to meet this need.

The UK is focusing on embedding clinical research within the NHS; the vision set out in 2021 was to create a research-positive culture, where all staff feel empowered and supported to participate in clinical research delivery as part of their job [30], NHS England Chief Officer’s strategy documents for Nurses [31], Midwives [32] and Allied Health Professionals [33] articulate that research is a part of the profession’s roles, and clinical trials feature in the recent government’s 10-year health plan [34]. With the drive to increase healthcare professionals’ engagement in research aiming to offer more research opportunities to patients, it must be considered that one consequence may be an increase in the number of patients and healthcare professionals who experience a premature trial termination.

Clinical research nurses and midwives are specialists in research delivery, with an element of the role ensuring participants receive the protocol required care and monitoring [35], and in the case of a premature trial termination, what is required is no longer clear. Premature trial termination has now been shown to be challenging and lacking guidance; healthcare professionals are therefore required to provide care and support without sufficient information, relevant training or guidance.

### Limitations

Additional data such as respondent’s role, clinical research involvement and their work location would have been useful to understand more about respondents’ context of practice. This may have impacted the decision to exclude three responses, as respondents did not identify themselves as healthcare professionals and were therefore excluded. Further demographic data might have provided insight into their role and therefore eligibility. However, the decision was made to keep demographic data to a minimum to reduce the questionnaire length as much as possible.

Electronic questionnaire was selected as the method to support national participation with minimal burden. However, the limitation of this was that there was no opportunity to prompt or clarify information, which would have ensured all answers were able to be understood.

The pilot group for the questionnaire was a convenience sample, and as such did not include all professions within the target population, which may have contributed to questions being open to misinterpretation. We acknowledge that we have reported challenges for patients/families, which are based on perceptions or direct feedback from trial participants/families to healthcare professionals, which leaves potential for misinterpretation.

A total of 709 respondents discontinued after reading the opening page, likely reflecting that they did not fulfil the eligibility criteria or did not feel they had it relevant to them. This reflects the challenge of reaching UK healthcare professionals with experience of the phenomenon of interest due to the diversity of roles and professions involved. Using social media as a recruitment strategy is known to be challenging [36], but this, alongside ‘snowballing’, was felt to be the best way to reach the wide range of healthcare professionals who had relevant experience. The study has a small sample size, which makes it difficult to report that these findings are generalisable; however, it does highlight a difficult situation that healthcare staff are experiencing and an area where improvements can be made.

### Future research

This study highlights that premature termination of clinical trials is a situation experienced by UK healthcare professionals that presents challenges for healthcare professionals, services, research participants and/or families. Future research is needed to explore the experience from the participants’ and family’s perspective and to determine which resources healthcare professionals find helpful to support them in this situation. Prematurely terminating clinical trials are not positively experienced, and further research has the potential to improve this for participants, families and healthcare professionals.

## Conclusion

This study shows that UK healthcare professionals delivering clinical research have experienced the premature termination of both pediatric and adult trials, and the situation is challenging for staff, services, participants and families. Those delivering and participating in research need to be aware of this potential event. Healthcare professionals should be skilled in recognising and managing emotional distress in both themselves and others. Clinical trial participants should feel appropriately supported if their clinical trial prematurely terminates, which will require healthcare professionals to be suitably prepared and trained.

## Supplementary Information

Supplementary Material 1: Question section from questionnaire.

Supplementary Material 2: Checklist for Reporting Of Survey Studies.

## Data Availability

The datasets used and/or analysed during the current study are available from the corresponding author on reasonable request.
