# Supplementary material for: Occurrence and management of premature clinical trial termination: a survey of UK healthcare professionals
Source: Trials. 2026 Jan 22;27:136. doi: 10.1186/s13063-026-09441-9 (PMC12910750; doi:10.1186/s13063-026-09441-9)
Supplement: Supplementary file 1 — Supplementary Material 1: Question section from questionnaire. [file 13063_2026_9441_MOESM1_ESM.docx]

**Supplementary Material – Question section from questionnaire.**

Clinical trial premature termination

Eligibility

Are you a UK healthcare professional who delivers clinical research as part of your role? *Required*

YES

NO

Consent

By consenting to take part you are confirming you understand the purpose of this

questionnaire and how your data will be used. You may choose to stop and exit the

questionnaire at any time. *Required*

I consent to take part

I do not consent to take part

About you

What is your profession? *Required*

Nurse

Doctor

Midwife

Physiotherapist

Pharmacist

Radiographer

Dietician

Occupational Therapist

Dentist

Other

If you selected Other, please specify: [Free text]

Do you work in the NHS? *Required*

YES

NO

If NO, which type of organisation do you work in? e.g. University [Free text]

Have you worked on any clinical trial(s) that has terminated prematurely? *Required*

YES – continues with next section ‘Your experience’

NO – continues with section ‘Resources’

Your experience

Was this with adult or paediatric participants or both? *Required*

Adult research participants

Paediatric Research Participants

Both

When the clinical trial prematurely terminated was a medication/device/therapy/treatment stopped for your participants? *Required*

YES

NO

Were there any particular issues that were challenging for you or the service? *Required* [Free text]

Were there any issues that were challenging for participants and/or their families?

*Required* [Free text]

Resources

In your workplace do you have any methods/tools/strategies/standard operating

procedures/protocols in place to manage the premature termination of clinical trials? *Required*

YES

NO

If YES, Please describe these resources *Required* [Free text]

If you are prepared and able to share these resources, we would like to review them to explore which topics they address.

Resources will be stored securely and information that identifies departments,

organisations or individuals will be held confidentially. You may prefer to remove

such information before sharing. The analysis and results will not identify the origin of the resources.

Resources can be emailed to [Researcher’s email address]. Please note that emailing will identify you to the researcher.

If you would rather not be linked to the resource or identified by email correspondence, you can send via post anonymously to:

[Researcher’s postal address]
